# Clinical scenarios of unusual FDG uptake in muscle

**DOI:** 10.1007/s11604-024-01672-7

**Published:** 2024-10-16

**Authors:** Ryogo Minamimoto, Katsuhiko Kato, Shinji Naganawa

**Affiliations:** 1 Department of Integrated Image Information Analysis, Nagoya University Graduate School of Medicine, 65, Tsurumaicho, Shouwa-ku, Nagoya, Aichi 466-8550 Japan; 2 Department of Radiology, Nagoya University Graduate School of Medicine, Nagoya, Aichi Japan; 3 Division of Advanced Information Health Sciences, Department of Integrated Health Sciences, Functional Medical Imaging, Biomedical Imaging Sciences, Nagoya, Aichi Japan

**Keywords:** FDG, PET, Muscle, Physiological, Glucose

## Abstract

Glucose is essential for muscle function and its uptake is influenced by aerobic conditions, hormonal regulations, and exercise. ^18^F-Fluorodeoxyglucose (FDG), a glucose analog used in PET/CT scans, can show incidental uptake in muscles, and thus careful interpretation is required to avoid misdiagnosis. Proper patient preparation and understanding of the clinical scenarios affecting FDG uptake are crucial for accurate PET/CT interpretation, thus ensuring precise diagnoses and avoiding unnecessary interventions. This review emphasizes the need to consider patient-specific factors in evaluating incidental FDG uptake in muscle.

## Introduction

Glucose is one of the primary energy sources for muscle cells, enabling them to perform various functions essential for movement, exercise, and overall health. Glucose is transported into the cells through glucose transporters 1–14 (GLUT-1 through GLUT-14). It then undergoes glycolysis in the cytoplasm to produce adenosine triphosphate. The process of glucose transportation is tissue-specific, as different GLUT isoforms are expressed in various organs, each regulated by distinct mechanisms [1].

Muscle activity significantly increases glucose consumption [2]. The uptake of glucose by muscles is influenced by factors such as aerobic conditions, hormonal regulations, and physical exercise [3]. GLUT4 is an insulin-responsive glucose transporter primarily expressed in skeletal muscle, as well as in the heart, adipose tissue, and the brain [1]. Plasma insulin does not directly increase glucose uptake but facilitates it by translocating GLUT 4 from intracellular vesicles to the plasma membrane, acting as a major gateway for glucose transport into cells. ^18^F-Fluorodeoxyglucose (FDG) is a glucose analog that is taken up by muscle cells via the same mechanism as glucose. Unlike glucose, however, FDG metabolism is halted within the cell, preventing its use for energy production through glycolysis or storage in muscle tissue [4].

Based on these regulations, FDG positron emission tomography/computed tomography (PET/CT) scans may show incidental uptake in muscles, making careful interpretation to distinguish physiological uptake from active lesions. This review highlights the diverse patterns of incidental FDG uptake in muscle, emphasizing the need to consider patient-specific factors to ensuring proper patient management.

## Diffuse FDG uptake in muscle

**Fig. 1:**
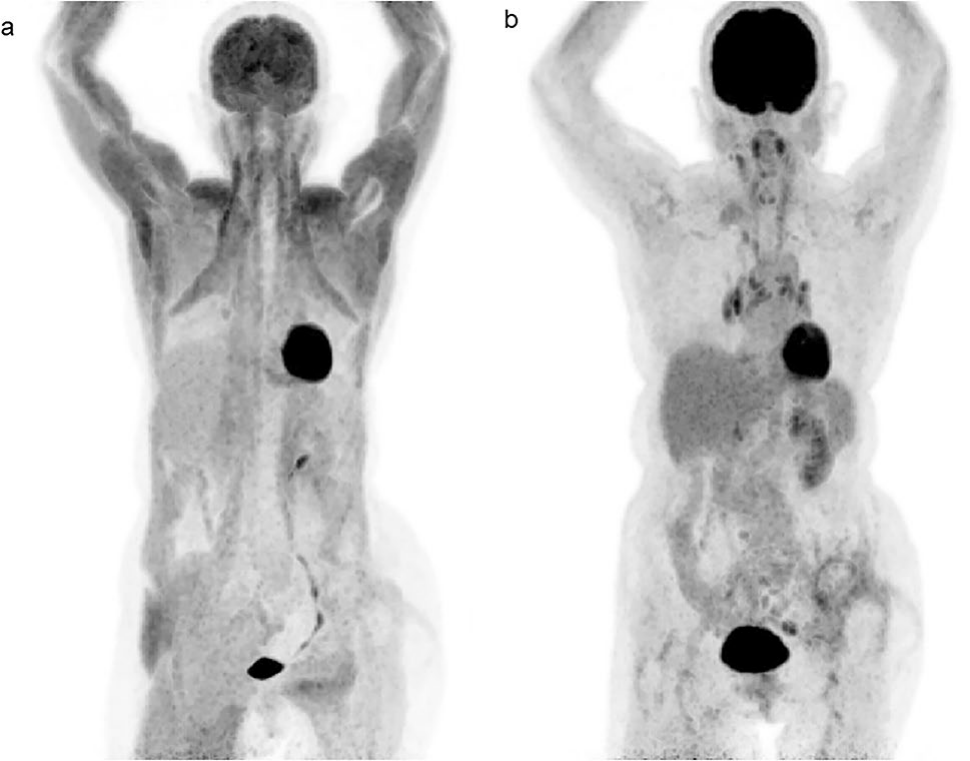
**a** A patient who had eaten a meal within 4 h from injection of FDG. **b** Restudy of FDG-PET/CT after one month in the same patient. Abnormal uptake in whole-body muscle and decreased FDG uptake in the brain were seen in (**a**), despite a BS level of 120 mg/dL at the time of FDG injection, has disappeared in the restudy (**b**). BS levels typically decrease to normal levels approximately two hours after the last food intake except in patients with diabetes. For this reason, it is necessary to conduct a detailed interview with the patient before the FDG injection, in addition to checking their BS level

FDG-PET/CT requires strict fasting for 4–6 h or more prior to scanning, but FDG uptake in muscle is not influenced by the time interval between FDG administration and the scan [5]. Administration or intake of glycemic agents leads to a diffuse increase in muscle FDG uptake and a reduction in uptake in the brain and viable lesions. Extensive skeletal muscle FDG accumulation can be caused by diabetes (also in suspected cases of diabetes), administration of insulin, and the presence of gastric food residue, even if fasting for at least 4 h before FDG administration [6]. In such cases, the FDG uptake pattern is typically symmetrical with mild to moderate intensity [7] (Fig. 1).

**Fig. 2:**
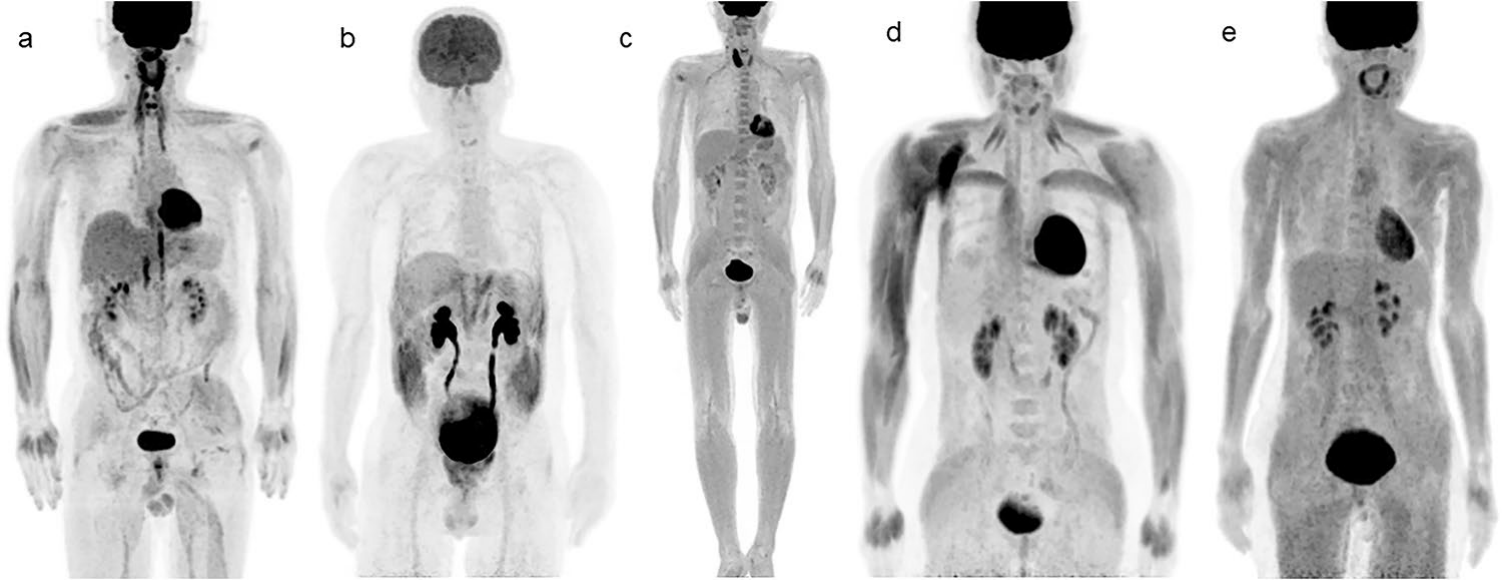
**a** Stress exercise before FDG injection. **b** Patient strained during a bowel movement. **c** Patients administered rapid-acting insulin before the injection of FDG. **d** Patient diagnosed with insulinoma, and **e** Patient diagnosed with Basedow’s disease. Abnormal FDG uptake in large muscles is confirmed in these specific situations

The rate of glucose metabolism in muscle is lower in diabetic patients. Accordingly, glucose uptake may vary with the progression of diabetes, showing an increase in states of hyperinsulinemia [8, 9]. A previous study demonstrated that subcutaneous administration of rapid-acting insulin compromised the quality of PET/CT studies if FDG was administered less than 4 h after the insulin (Fig. 2). The average SUV mean in the proximal middle third of the rectus femoris muscle of the right thigh was 1.98 in patients with blood glucose levels over 168 mg/dL, following subcutaneous administration of rapid-acting insulin to lower blood glucose levels to 160 mg/dL or less. This value was higher compared to 1.15 in the control group [10]

In a study that administered rapid-acting insulin at 90 min before FDG injection in diabetic patients with fasting blood glucose (FBG) levels over 160 mg/dL, the average SUV_max_ of FDG in muscle was 1.0 ± 0.3. In comparison, that of non-diabetic patients with normal FBG levels below 100 mg/dL was 0.6 ± 0.2. In the diabetic patient group, visual interpretations ranged from normal biodistribution to mild muscular uptake [11].

Various types of insulin are currently used in the clinical setting. The EANM Procedure Guidelines for Tumour Imaging: Version 2.0 recommend that FDG injection should be administered no sooner than 4 h after the subcutaneous administration of rapid-acting insulin, and no sooner than 6 h after the administration of short-acting insulin. They also recommend that intermediate or long-acting insulin should not be administered on the day of the scan, with insulin being administered no earlier than the evening before. For insulin pumps that work as continuous infusions, the device should be switched off for a minimum of 4 h before FDG injection [12].

For insulin pumps that infuse rapid-acting insulin continuously, the amount of insulin is calculated based on the total daily dose of injected insulin. The insulin dose provided by the pump is estimated to meet the basal insulin need unless the patient uses the bolus infusion option integrated into the pump [13, 14]. In our experience, the continuous operation of insulin pumps around the time of an FDG-PET/CT study has less impact on FDG uptake. The administration of insulin (except rapid-acting insulin with short intervals from FDG administration) does not have a critical effect on image quality but can cause increased FDG uptake in muscles that can be evaluated quantitatively, which has potential utility in the diagnosis of lesions in the body.

The use of steroids within several months before PET can cause extensive skeletal muscle uptake regardless of blood glucose level [15]. Diffuse FDG uptake in muscles can also be seen in conditions such as malignancy [16], insulinoma [17], Basedow disease [18], and myositis [19], necessitating careful consideration during image interpretation (Fig. 2).

## Conscious or unconscious of vigorous exercise

Vigorous exercise results in increased glucose uptake in muscles. Exercise and endurance training in particular increase the rate of metabolism of glucose in skeletal muscle [9] (Fig. 2). It is recommended to avoid vigorous exercise for at least 24 h before an FDG-PET/CT examination, as its effects can persist for up to 48 h post-exercise [20–22]. Casual activities can also lead to increased muscle FDG uptake. Writing or turning pages of a book around the time of FDG injection can result in increased FDG uptake in the muscles of the forearm and hand [22], which can also be seen in subjects who use mobile devices for more than 60 min within 24 h before an FDG-PET/CT study [23]. The mode of transportation used to visit the hospital can result in characteristic patterns of muscle uptake [24]. We have observed FDG uptake in the hands and forearms of patients using wheelchairs, as well as in the thigh muscles of patients who cycled to their scan appointment.

**Fig. 3:**
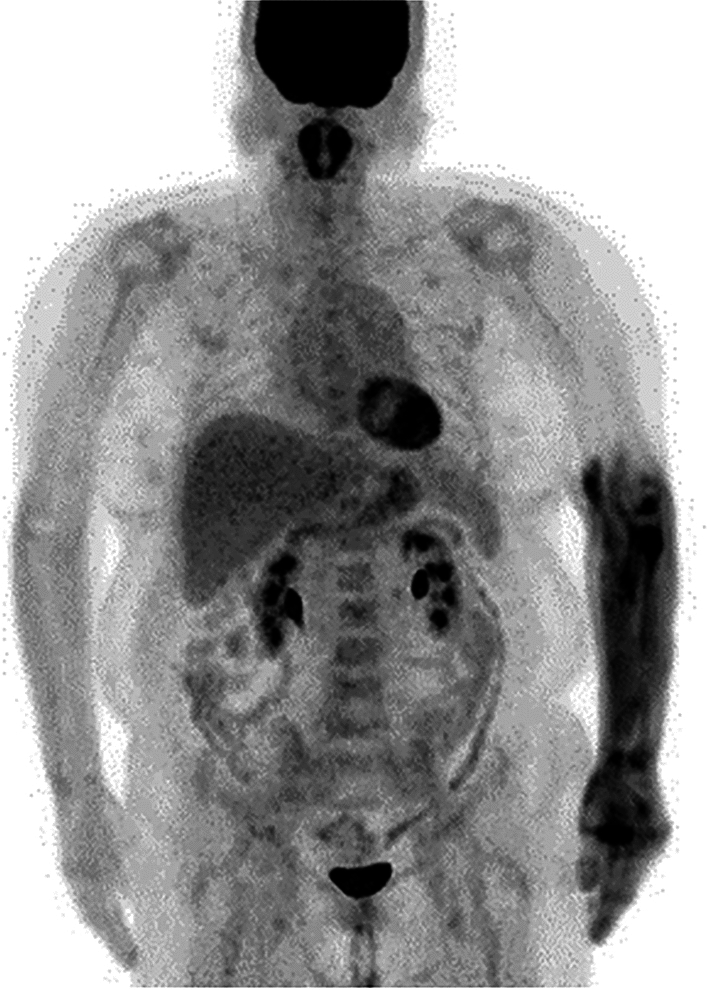
Hot forearm sign

FDG uptake in the forearm that is observed following intra-arterial injection is termed “hot forearm” or “hot hand” sign [25] (Fig. 3). A recent report also indicated that scratching or itching can show the same uptake pattern [26].

**Fig. 4:**
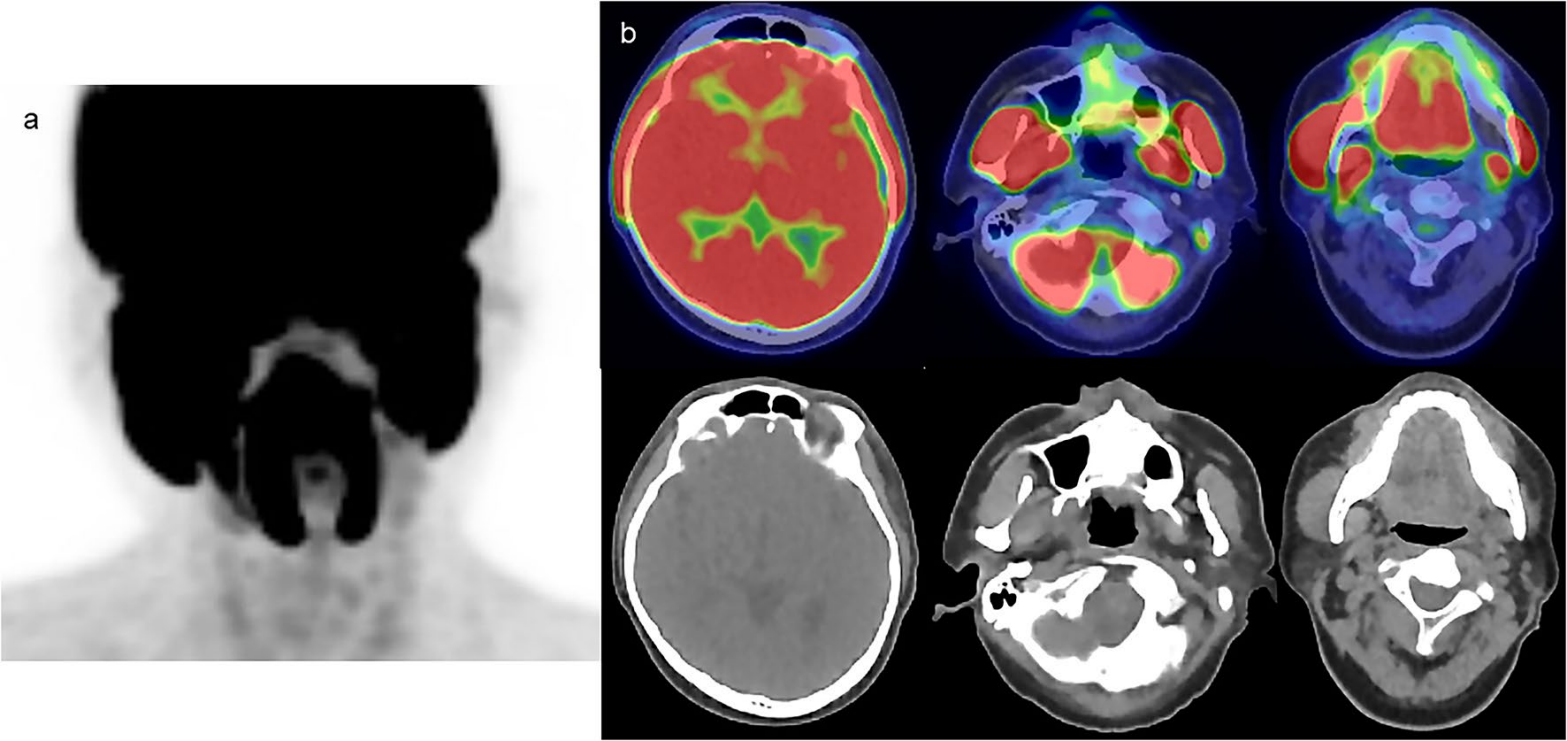
**a** FDG MIP image in a patient with Sjögren’s syndrome. **b** Upper: fusion images, lower: CT images. The high FDG uptake seen in the facial and masseter muscles is due to chewing gum

Chewing behavior increases uptake in the masseter, temporalis, and pterygoid muscles. Such behavior can occur habitually and should be avoided around the FDG-PET/CT examination [7]. However, it may be indicated to use such situations to purposely induce salivary secretion such as Sjögren’s syndrome [27] (Fig. 4). FDG uptake can occur in the facial muscles due to involuntary movements in patients with facial dyskinesia [28]. Several studies have reported that patients with cranial dystonia and blepharospasm dystonia show hypermetabolism in the basal ganglia, thalamus, and cerebellum [29, 30]. Speech can lead to increased FDG uptake in the bilateral laryngeal muscles [31]. Asymmetric FDG uptake in the laryngeal muscles (vocal cords) can indicate malignancy, vocal cord palsy [32], or conditions such as Reinke’s edema [33]. However, weak asymmetrical FDG uptake in the laryngeal muscles can also be observed in healthy individuals. Symptoms such as hoarseness and a review of the patient’s medical history can aid in differentiating these conditions.

**Fig. 5:**
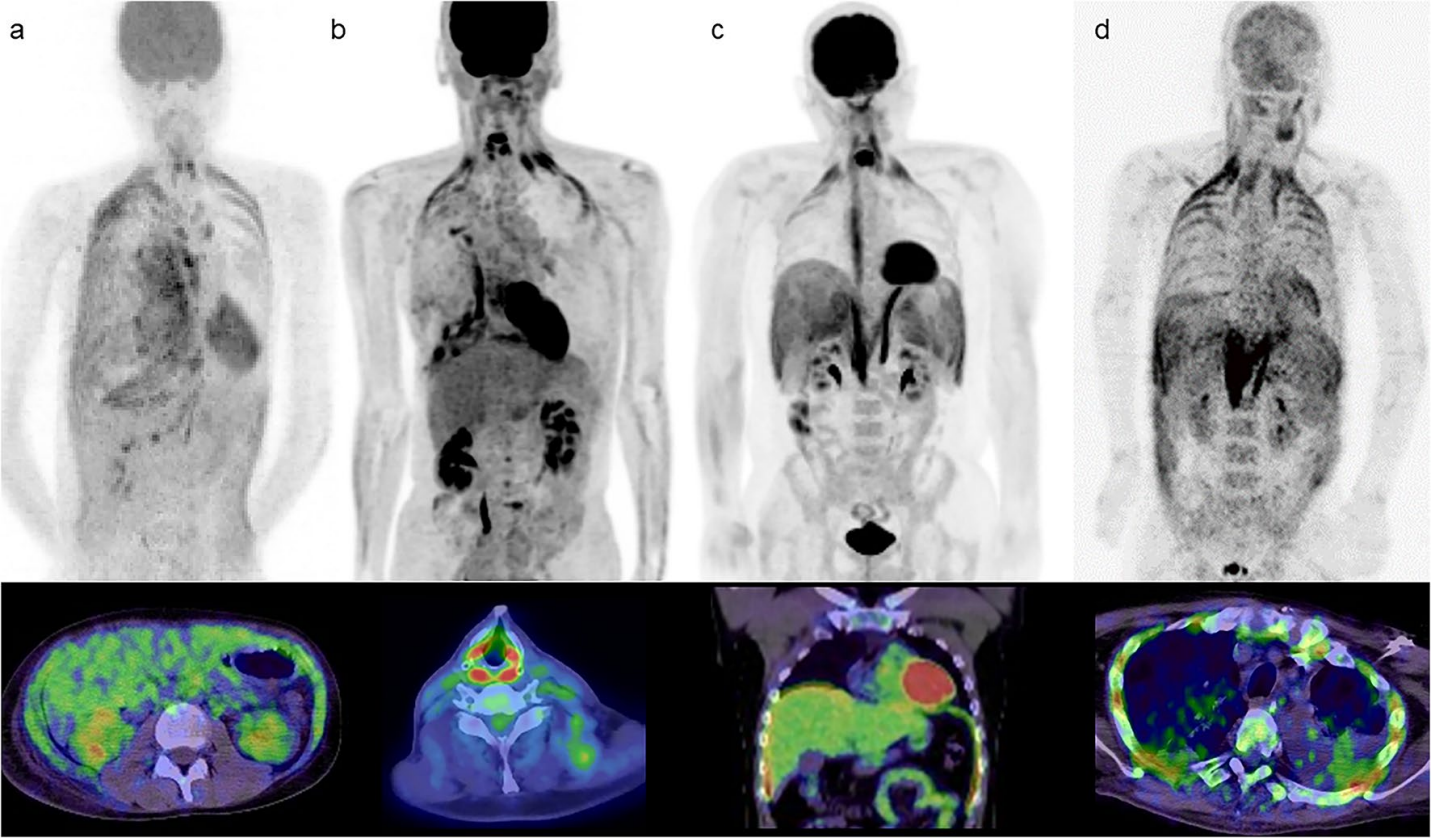
Images of patients with **a** dyspnea caused by a malignant pleural lesion, **b** frequent cough, **c** frequent hiccups, and **d** breathing supported by Continuous Positive Airway Pressure (CPAP). The cervical, sternocleidomastoid, intercostal, diaphragmatic, and abdominal muscles all work to support breathing. Uptake in the vocal cords is confirmed in the patient with frequent coughing

Increased FDG uptake in the genioglossus muscle has been seen in patients who have used a pacifier [34]. Stressful breathing and coughing can lead to significantly increased uptake in the scalene, sternocleidomastoid, and intercostal muscles [35] (Fig. 5). In more severe cases, labored breathing can also involve the diaphragm and abdominal muscles [36, 37] (Fig. 5). FDG uptake in respiratory muscles is correlated with chronic obstructive pulmonary disease [38]. Specifically, FDG uptake in the intercostal muscles is related to forced expiratory volume in 1 s/forced vital capacity (FEV1/FVC), as opposed to uptake in the neck or abdominal muscles [39].

## Focal FDG uptake in muscle

Focal FDG uptake in muscles occurs regularly in clinical situations, but medical procedures should also be taken into account. FDG uptake in the shoulder is a common finding in FDG-PET/CT scans. FDG accumulation in the teres minor muscle and muscles near the radioulnar joint by the elbow is often related to the side of tracer injection [40, 41]. FDG uptake in the rotator interval or inferior capsule of the shoulder, close to the teres minor muscle, is associated with adhesive capsulitis. The degree of uptake correlates with symptom severity and should be reported [42].

**Fig. 6:**
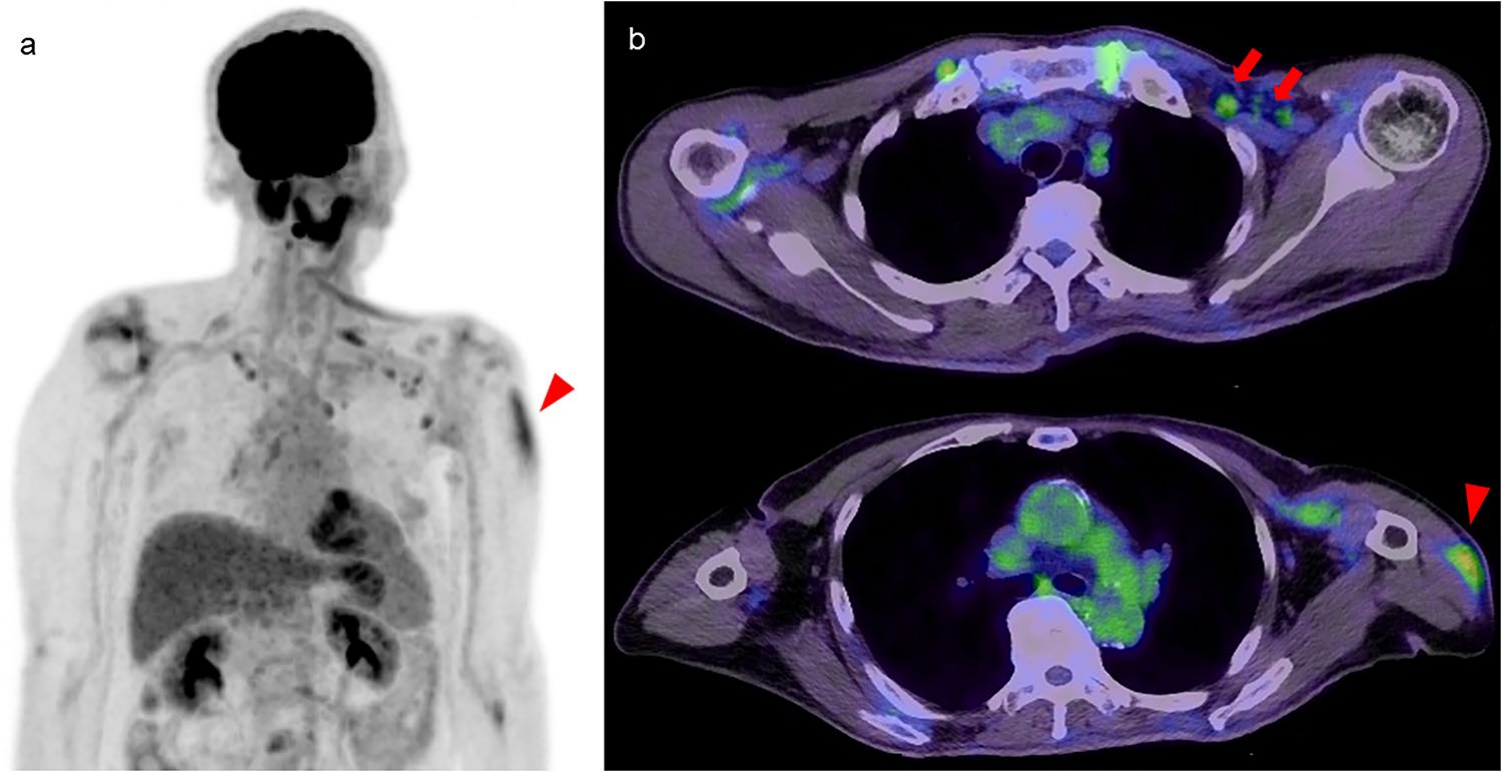
**a** FDG-PET MIP image. **b** Axial FDG-PET/CT images. Moderate FDG uptake is confirmed in the deltoid muscle (arrowhead), indicating inflammatory change following administration of the COVID-19 vaccine. FDG uptake in axillary lymph nodes can also be confirmed with this procedure (arrow)

**Fig. 7:**
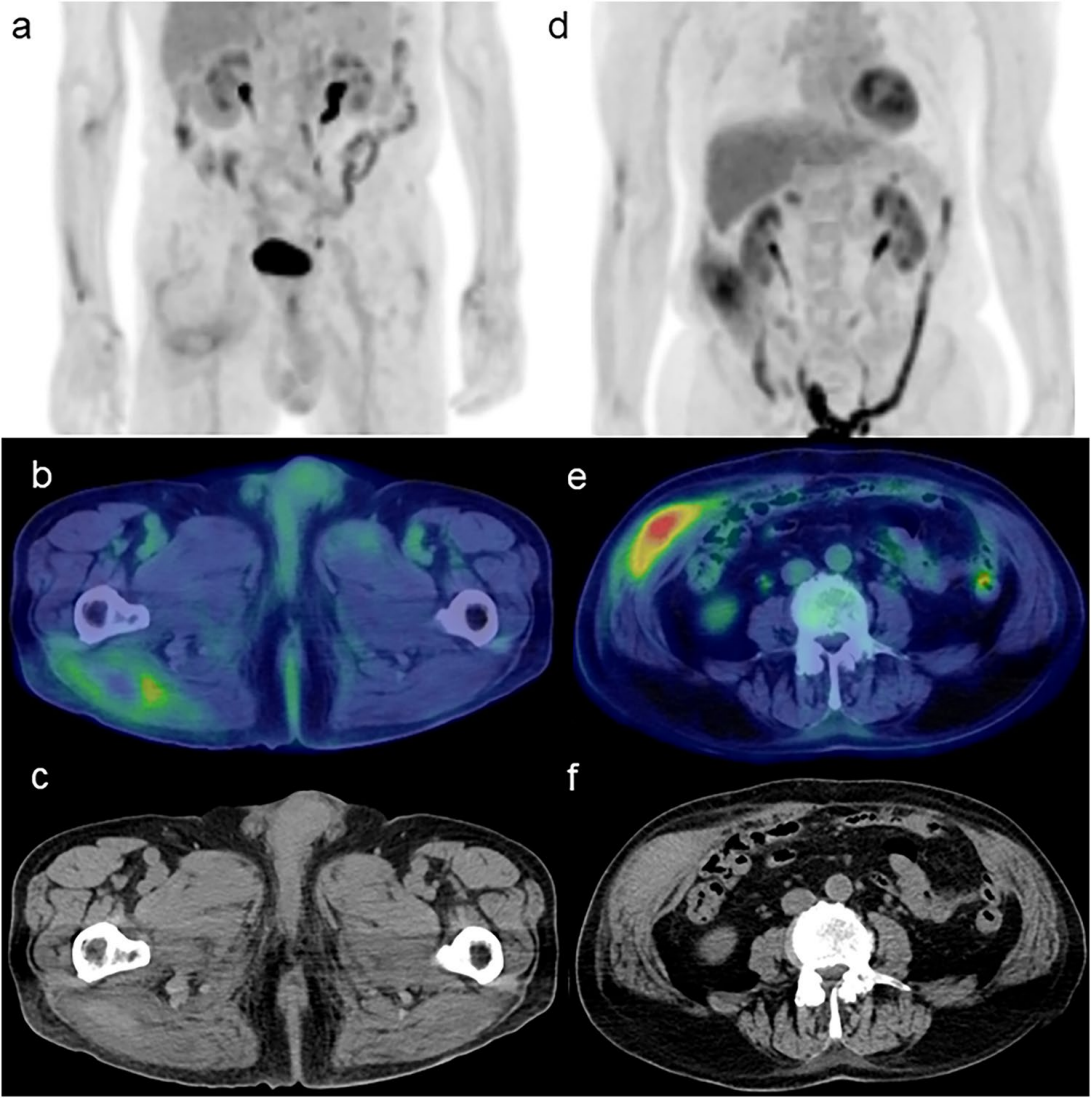
**a**, **d** FDG-PET MIP images. **b**, **e** Axial FDG-PET/CT images. **c**, **f** CT images. The region of high FDG uptake in swollen muscle corresponds to the hematoma apparent on CT as a region of relatively high attenuation

**Fig. 8:**
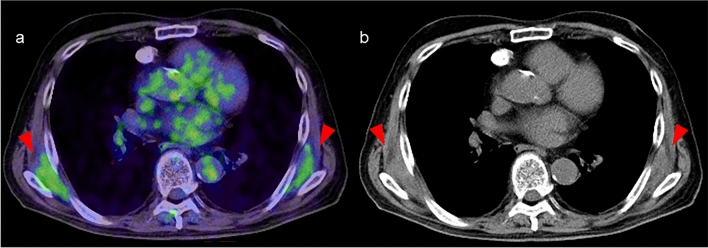
**a** Axial image of FDG-PET/CT and **b** CT image. Elastofibroma dorsi is typically characterized by mild to moderate FDG uptake, seen here in a soft tissue mass located around the bilateral inferior scapular angles (arrowhead)

Moderate FDG uptake has been confirmed in broad areas following the administration of the COVID-19 vaccine, indicating a high degree of inflammatory change in the deltoid muscle (Fig. 6). In these cases, FDG uptake in axillary lymph nodes can indicate the procedure [43]. Conversely, muscle uptake is not generally observed following subcutaneous administration (as is recommended in Japan) of the influenza vaccination, although there is a possibility of weak FDG uptake in axillary lymph nodes near the injection site [43]. FDG uptake in swollen muscle should prompt consideration of infection or abscess and may indicate hematoma in the case of focal and round or ring-like uptake (Fig. 7). Elastofibroma dorsi, a soft-tissue pseudotumor arising from mesenchymal tissue, can sometimes mimic a muscle lesion. It typically presents with mild to moderate (occasionally high) FDG uptake in a soft tissue mass located around the bilateral inferior scapular angles [44] (Fig. 8).

## Asymmetric and unilateral FDG uptake in muscle

**Fig. 9:**
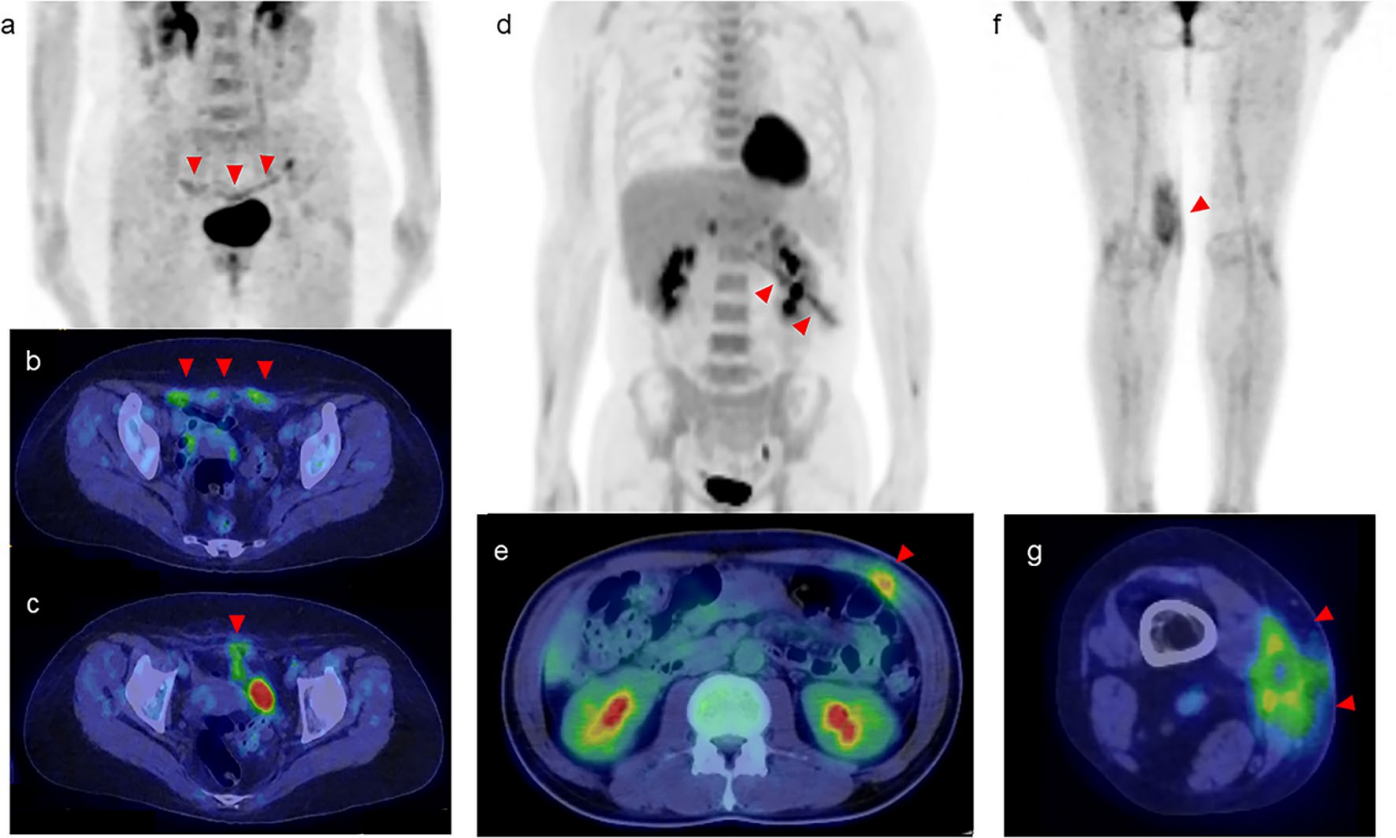
**a**, **d**, and **f** FDG-PET MIP images. **b**, **c**, **e**, and **g** Axial FDG-PET/CT images. Various degrees of FDG uptake (arrowheads) are seen, consistent with the surgical procedure in a short time since the surgery

**Fig. 10:**
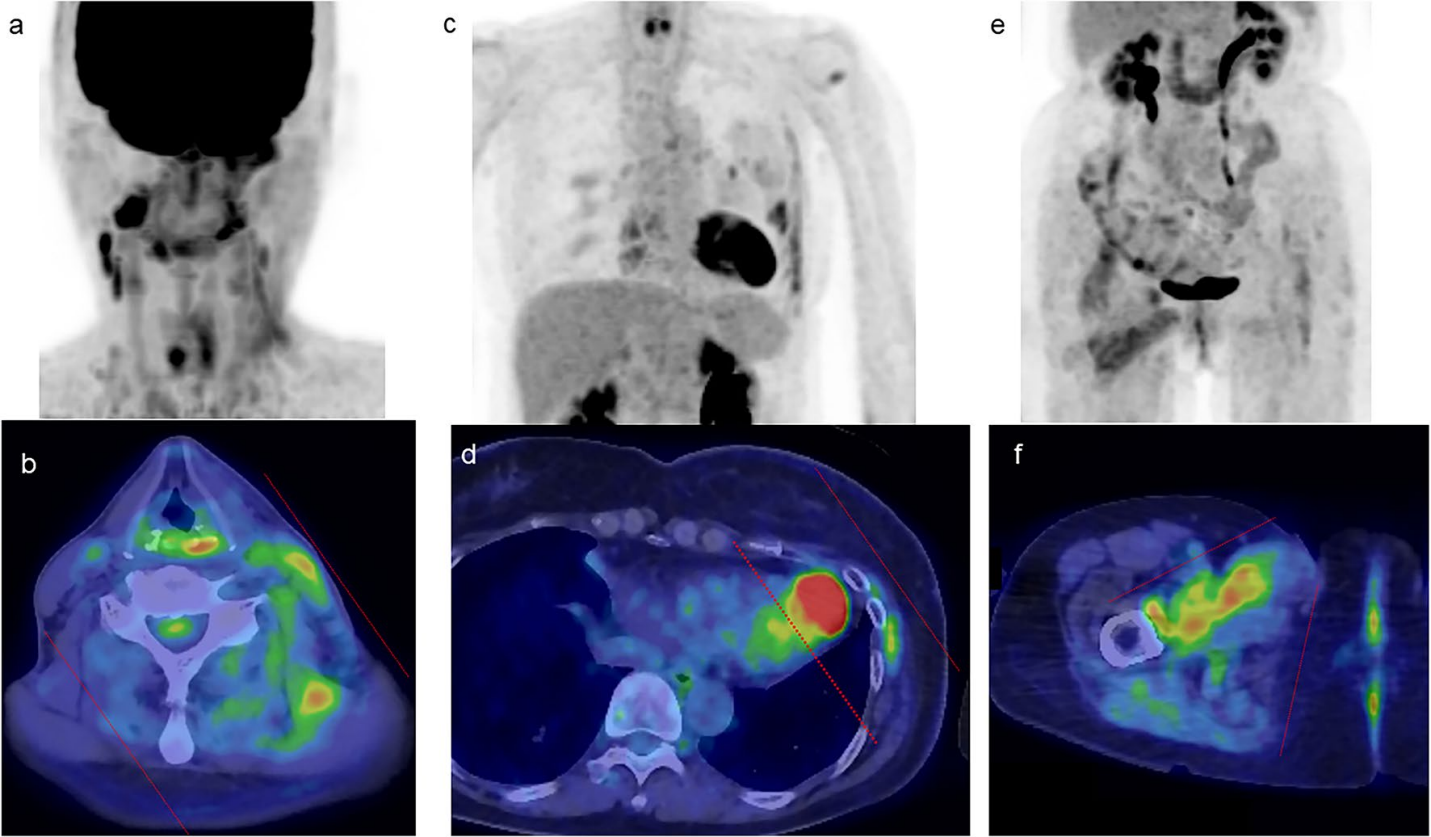
**a**, **c**, and **e** FDG-PET MIP images. **b**, **d**, and **f** Axial FDG-PET/CT images. The area of FDG uptake matches the field of radiation therapy (marked by dotted lines)

Asymmetric and unilateral muscle uptake can be either physiologic or pathologic. The various clinical conditions and interventions usually lead to linear and mild to moderate FDG intensity in muscles, but uptake can sometimes be high [7]. Linear FDG uptake in muscle is commonly observed after open surgery and typically converges into focal uptake over time (Fig. 9). When linear uptake is observed, it is important to carefully assess the FDG-PET/CT images because the uptake may be related to the surgical procedure, potentially leading to false positive findings. FDG uptake around a lesion and/or segmental uptake can reflect localized inflammation within the radiotherapy field [45] (Fig. 10). This type of muscle uptake can persist for up to 18 months post-radiotherapy [22]. In addition, regional FDG uptake observed in muscle can be due to inflammatory changes related to trauma.

Denervated muscles with peripheral nerve injury, confirmed by electromyography, show higher FDG uptake than normal muscles. This is associated with the severity of the injury and traumatic etiology [46, 47]. Previous studies have indicated that neuropathy caused by non-traumatic injuries such as tumor invasion and radiation therapy progresses more slowly than those caused by traumatic injuries [46]. Increased postoperative FDG uptake in the cervical muscles was often observed on the dissected side following neck dissection, likely due to denervation [48].

Consequently, FDG uptake in the affected area tends to be lower than that on the healthy side. Various other types of FDG uptake in muscle are commonly observed in routine FDG-PET scans, many of which may be attributed to a specific cause [49]. To differentiate between FDG physiologic uptake in skeletal muscle and uptake in pathologic involvement, proper patient preparation is essential.

## Conclusion

It is crucial to distinguish physiological uptake from active lesions and to consider the specific conditions of the subject. Accurate interpretation of FDG-PET scans requires a comprehensive understanding of the potential causes of muscle FDG uptake, including physical activity, inflammation, and recent medical procedures, to avoid misdiagnosis and ensure proper patient management.

## References

[1] Navale AM, Paranjape AN. Glucose transporters: physiological and pathological roles. Biophys Rev. 2016;8:5–9. 28510148 10.1007/s12551-015-0186-2 PMC5425736

[2] Felig P, Wahren J. Fuel homeostasis in exercise. N Engl J Med. 1975;293:1078–84. 1178025 10.1056/NEJM197511202932107

[3] Richter EA, Hargreaves M. Exercise, GLUT4, and skeletal muscle glucose uptake. Physiol Rev. 2013;93:993–1017. 23899560 10.1152/physrev.00038.2012

[4] Gambhir SS. Molecular imaging of cancer with positron emission tomography. Nat Rev Cancer. 2002;2:683–93. 12209157 10.1038/nrc882

[5] Wang R, Chen H, Fan C. Impacts of time interval on ^18^F-FDG uptake for PET/CT in normal organs: a systematic review. Medicine (Baltimore). 2018;97: e13122. 30407330 10.1097/MD.0000000000013122 PMC6250532

[6] Cline GW, Petersen KF, Krssak M, Shen J, Hundal RS, Trajanoski Z, et al. Impaired glucose transport as a cause of decreased insulin-stimulated muscle glycogen synthesis in type 2 diabetes. N Engl J Med. 1999;341:240–6. 10413736 10.1056/NEJM199907223410404

[7] Liu Y, Ghesani NV, Zuckier LS. Physiology and pathophysiology of incidental findings detected on FDG-PET scintigraphy. Semin Nucl Med. 2010;40:294–315. 20513451 10.1053/j.semnuclmed.2010.02.002

[8] Nakatani K, Nakamoto Y, Togashi K. Risk factors for extensive skeletal muscle uptake in oncologic FDG-PET/CT for patients undergoing a 4-h fast. Nucl Med Commun. 2012;33:648–55. 22395030 10.1097/MNM.0b013e328352290f

[9] Selberg O, Müller MJ, van den Hoff J, Burchert W. Use of positron emission tomography for the assessment of skeletal muscle glucose metabolism. Nutrition. 2002;18:323–8. 11934545 10.1016/s0899-9007(01)00799-7

[10] Garcia JR, Sanchis A, Juan J, Tomas J, Domenech A, Soler M, et al. Influence of subcutaneous administration of rapid-acting insulin in the quality of ^18^F-FDG PET/CT studies. Nucl Med Commun. 2014;35:459–65. 24535382 10.1097/MNM.0000000000000082

[11] Rallapeta RP, Manthri RG, Kalawat T, Sachan A, Lakshmi AY, Hulikal N. Utility of short-acting intravenous insulin therapy in preparation of F-18 fluorodeoxyglucose positron emission tomography computed tomography scan in cancer patients incidentally detected with high blood glucose levels on the day of test. Indian J Nucl Med. 2020;35:110–5. 32351264 10.4103/ijnm.IJNM_151_19 PMC7182326

[12] Boellaard R, Delgado-Bolton R, Oyen W, Giammarile F, Tatsch K, Eschner W, et al. FDG PET/CT: EANM procedure guidelines for tumour imaging: version 2.0. Eur J Nucl Med Mol Imaging. 2015;42:328–54. 25452219 10.1007/s00259-014-2961-x PMC4315529

[13] Thomas SJ. Crabtree, Anastasios Gazis, Insulin pumps and diabetes technologies in pregnancy: an overview for the obstetrician. Obstet Gynaecol Reprod Med. 2020;30(4):126–9.

[14] Paldus B, Lee MH, O’Neal DN. Insulin pumps in general practice. Aust Prescr. 2018;41:186–90. 30670886 10.18773/austprescr.2018.056 PMC6299172

[15] Kawabata K, Hosono M, Mori Y, Tsukamoto S, Ito S, Ando S, et al. Steroids may be associated with extensive skeletal muscle uptake of ^18^F-FDG. Clin Nucl Med. 2023;48:1015–20. 37756474 10.1097/RLU.0000000000004856

[16] Broski SM, Bou-Assaly W, Gross MD, Fig LM. Diffuse skeletal muscle [^18^F] fluorodeoxyglucose uptake in advanced primary muscle non-Hodgkin’s lymphoma. Clin Nucl Med. 2009;34:251–3. 19300063 10.1097/RLU.0b013e31819a2020

[17] Kamaleshwaran KK, Bhattacharya A, Chakraborty D, Manohar K, Mittal BR. Diffusely increased muscular uptake of [^18^F]fluorodeoxyglucose: a clue for the diagnosis of insulinoma? Eur J Nucl Med Mol Imaging. 2010;37:1800. 20596865 10.1007/s00259-010-1515-0

[18] Chen YK, Chen YL, Liao AC, Shen YY, Kao CH. Elevated ^18^F-FDG uptake in skeletal muscles and thymus: a clue for the diagnosis of Graves’ disease. Nucl Med Commun. 2004;25:115–21. 15154698 10.1097/00006231-200402000-00004

[19] Rixey AB, Glazebrook KN, Powell GM, Baffour FI, Collins MS, Takahashi EA, et al. Rhabdomyolysis: a review of imaging features across modalities. Skeletal Radiol. 2024;53:19–27. 37318587 10.1007/s00256-023-04378-5

[20] Gradinscak DJ, Fulham MJ, Mohamed A, Constable CJ. Skeletal muscle uptake detected on FDG PET 48 hours after exertion. Clin Nucl Med. 2003;28:840–1. 14508278 10.1097/01.rlu.0000090932.84059.80

[21] Abouzied MM, Crawford ES, Nabi HA. ^18^F-FDG imaging: pitfalls and artifacts. J Nucl Med Technol. 2005;33:145–55. 16145222

[22] Parida GK, Roy SG, Kumar R. FDG-PET/CT in skeletal muscle: pitfalls and pathologies. Semin Nucl Med. 2017;47:362–72. 28583276 10.1053/j.semnuclmed.2017.02.003

[23] Bai X, Wang X, Zhuang H. Relationship between the elevated muscle FDG uptake in the distal upper extremities on PET/CT scan and prescan utilization of mobile devices in young patients. Clin Nucl Med. 2018;43:168–73. 29293133 10.1097/RLU.0000000000001967

[24] Okuyama C, Kusano K, Ito M, Takase A, Goda S, Kagawa S. Characteristic muscular FDG uptake patterns related to the transportation means used by patients to visit the hospital. Clin Nucl Med. 2023;48:549–52. 36928161 10.1097/RLU.0000000000004622

[25] Kumar K. Abnormally increased uptake of 18F-FDG in the forearm and hand following intra-arterial injection–hot forearm and hot hand signs. Br J Radiol. 2009;82:995–9. 19470569 10.1259/bjr/62898427 PMC3473382

[26] Kumar SA, Mittal BR, Sindhu T, Kumar R. “Bilateral Hot Forearm Sign”: ingeminating the pattern of physiological uptake of ^18^F-fludeoxyglucose. Indian J Nucl Med. 2024;39:61–2. 38817722 10.4103/ijnm.ijnm_145_23 PMC11135376

[27] Lee DH, Yoon JK, Yoon SH, Lee SJ, An YS. Physiologic facial muscle uptake on ^18^F-FDG PET/CT by chewing-like habitual movement in patient with Sjögren syndrome. Clin Nucl Med. 2015;40:268–9. 25608147 10.1097/RLU.0000000000000683

[28] Sung DH, Choi JY, Kim DH, Kim ES, Son YI, Cho YS, et al. Localization of dystonic muscles with ^18^F-FDG PET/CT in idiopathic cervical. J Nucl Med. 2007;48:1790–5. 17942812 10.2967/jnumed.107.044024

[29] Esmaeli-Gutstein B, Nahmias C, Thompson M, Kazdan M, Harvey J. Positron emission tomography in patients with benign essential blepharospasm. Ophthalmic Plast Reconstr Surg. 1999;15:23–7. 9949425 10.1097/00002341-199901000-00006

[30] Hutchinson M, Nakamura T, Moeller JR, Antonini A, Belakhlef A, Dhawan V, et al. The metabolic topography of essential blepharospasm: a focal dystonia with general implications. Neurology. 2000;55:673–7. 10980732 10.1212/wnl.55.5.673

[31] Kostakoglu L, Wong JC, Barrington SF, Cronin BF, Dynes AM, Maisey MN. Speech-related visualization of laryngeal muscles with fluorine-18-FDG. J Nucl Med. 1996;37:1771–3. 8917172

[32] Minamimoto R, Kubota K, Morooka M, Ito K, Mitsumoto T, Okasaki M, et al. Reevaluation of FDG-PET/CT in patients with hoarseness caused by vocal cord palsy. Ann Nucl Med. 2012;26:405–11. 22427268 10.1007/s12149-012-0588-1

[33] Erdoğan O, İsmi O, Arpacı RB, Vayısoğlu Y, Özcan C. False-positive laryngeal FDG uptake during PET/CT imaging: Reinke’s Edema. Turk Arch Otorhinolaryngol. 2018;56:114–6. 30197811 10.5152/tao.2018.3123 PMC6123119

[34] Tong C, Zhuang H. Increased genioglossus muscle FDG activity due to using pacifier. Clin Nucl Med. 2022;47:655–7. 35195586 10.1097/RLU.0000000000004105

[35] Legrand A, Schneider E, Gevenois P, De Troyer A. Respiratory effects of the scalene and sternomastoid muscles in humans. J Appl Physiol. 2003;94:1467–72. 12626472 10.1152/japplphysiol.00869.2002

[36] Aydin A, Hickeson M, Yu JQ, Zhuang H, Alavi A. Demonstration of excessive metabolic activity of thoracic and abdominal muscles on FDG-PET in patients with chronic obstructive pulmonary disease. Clin Nucl Med. 2005;30:159–64. 15722818 10.1097/00003072-200503000-00003

[37] Okabayashi H, Machida H, Masunaga A, Ichiyasu H, Sakagami T. ^18^F-FDG uptake in accessory respiratory muscles shows the respiratory effort of patients with pleuroparenchymal fibroelastosis. Respirol Case Rep. 2022;10: e0900. 35079403 10.1002/rcr2.900 PMC8776897

[38] Osman MM, Tran IT, Muzaffar R, Parkar N, Sachdeva A, Ruppel GL. Does ^1^⁸F-FDG uptake by respiratory muscles on PET/CT correlate with chronic obstructive pulmonary disease? J Nucl Med Technol. 2011;39:252–7. 22082614 10.2967/jnmt.111.089961

[39] Kothekar E, Borja AJ, Gerke O, Werner TJ, Alavi A, Revheim ME. Assessing respitatory muscle activity with ^18^F-FDG-PET/CT in patients with COPD. Am J Nucl Med Mol Imaging. 2019;9:309–15. 31976160 PMC6971478

[40] Nakatani K, Nakamoto Y, Togashi K. Unilateral physiological FDG uptake in teres minor muscle seems well associated with IV tracer injection procedures. Clin Nucl Med. 2015;40:62–4. 24662656 10.1097/RLU.0000000000000406

[41] Otomi Y, Shinya T, Uyama N, Arai Y, Miyamoto K, Takechi K, et al. The physiological accumulation of FDG in the muscles in relation to the side of intravenous administration. Jpn J Radiol. 2017;35:53–60. 27812958 10.1007/s11604-016-0597-4

[42] Hayashi D, Gould E, Shroyer R, van Staalduinen E, Yang J, Mufti M, et al. Shoulder adhesive capsulitis in cancer patients undergoing positron emission tomography—computed tomography and the association with shoulder pain. World J Radiol. 2021;13:344–53. 34786189 10.4329/wjr.v13.i10.344 PMC8567438

[43] Minamimoto R, Kiyomatsu T. Effects of COVID-19 vaccination on FDG-PET/CT imaging: a literature review. Glob Health Med. 2021;3:129–33. 34250287 10.35772/ghm.2021.01076 PMC8239370

[44] Fang N, Wang YL, Zeng L, Wu ZJ, Cui XJ, Wang Q, et al. Characteristics of elastofibroma dorsi on PET/CT imaging with ^18^F-FDG. Clin Imaging. 2016;40:110–3. 26432711 10.1016/j.clinimag.2015.08.009

[45] Okuyama C, Higashi T, Ishizu K, Saga T. FDG-PET findings associated with various medical procedures and treatments. Jpn J Radiol. 2023;41:459–76. 36575286 10.1007/s11604-022-01376-w PMC9794480

[46] Choi JS, Seo HG, Oh BM, Choi H, Cheon GJ, Lee SU, et al. ^18^F-FDG uptake in denervated muscles of patients with peripheral nerve injury. Ann Clin Transl Neurol. 2019;6:2175–85. 31588693 10.1002/acn3.50899 PMC6856607

[47] Pak K, Shin MJ, Hwang SJ, Shin JH, Shin HK, Kim SJ, et al. Longitudinal changes in glucose metabolism of denervated muscle after complete peripheral nerve injury. Mol Imaging Biol. 2016;18:741–7. 27028758 10.1007/s11307-016-0948-7

[48] Iritani Y, Kato H, Kaneko Y, Ishihara T, Ando T, Kawaguchi M, et al. FDG uptake in the cervical muscles after neck dissection: imaging features and postoperative natural course on ^18^F-FDG-PET/CT. Jpn J Radiol. 2024;42:892–8. 38658502 10.1007/s11604-024-01568-6 PMC11286666

[49] Yamane T, Matsusaka Y, Fukushima K, Seto A, Matsunari I, Kuji I. Atlas of non-pathological solitary or asymmetrical skeletal muscle uptake in [^18^F] FDG-PET. Jpn J Radiol. 2022;40:755–67. 35344131 10.1007/s11604-022-01264-3 PMC9345840

